# Two onset types of achalasia and the long-term course to diagnosis

**DOI:** 10.1007/s10388-024-01069-5

**Published:** 2024-06-06

**Authors:** Akane Kurosugi, Tomoaki Matsumura, Michiko Sonoda, Tatsuya Kaneko, Satsuki Takahashi, Kenichiro Okimoto, Naoki Akizue, Yuhei Ohyama, Yukiyo Mamiya, Hayato Nakazawa, Ryosuke Horio, Chihiro Goto, Yuki Ohta, Takashi Taida, Atsuko Kikuchi, Mai Fujie, Kentaro Murakami, Masaya Uesato, Yoshihito Ozawa, Jun Kato, Hisahiro Matsubara, Naoya Kato

**Affiliations:** 1 Department of Gastroenterology, Graduate School of Medicine, Chiba University, Inohana 1-8-1, Chiba, 260-8670 Japan; 2 Department of Frontier Surgery, Graduate School of Medicine, Chiba University, Chiba, Japan; 3 Department of Clinical Engineering Center, Chiba University Hospital, Chiba, Japan; 4 Clinical Research Center, Chiba University, Chiba, Japan

**Keywords:** Esophageal achalasia, High-resolution manometry, Anhidrosis

## Abstract

**Background:**

Recently, the incidence of achalasia has been increasing, but its cause remains unknown. This study aimed to examine the initial symptoms and the course of symptoms and to find new insights into the cause and course of the disease.

**Methods:**

Altogether, 136 patients diagnosed with achalasia by high-resolution manometry (HRM) were enrolled. Questionnaires and chart reviews were conducted to investigate the initial symptoms, time from onset to diagnosis, and comorbidities, as well as the relationship between HRM results, time to diagnosis, and symptom severity.

**Results:**

In total, 67 of 136 patients responded to the questionnaire. The median ages of onset and diagnosis were 42 and 58 years, respectively. The median time from onset to diagnosis was 78.6 months, with 25 cases (37.3%) taking > 10 years to be diagnosed. The symptom onset was gradual and sudden in 52 (77.6%) and 11 (16.4%) patients, respectively. Of the 11 patients with acute onset, three (27.3%) developed anhidrosis at the same time. There was no correlation between the time from onset to diagnosis and esophageal dilatation, resting LES pressure, or mean integrated relaxation pressure (IRP). No correlation was also found between the degree of symptoms and resting LES pressure or IRP.

**Conclusion:**

Esophageal achalasia can have acute or insidious onsets. This finding may help to elucidate the cause of achalasia.

## Introduction

Achalasia is an esophageal motility disorder associated with impaired relaxation and peristalsis of the lower esophageal sphincter (LES) [1]. Its annual incidence is approximately 1.6 cases per 100,000 persons, with a prevalence of 10 cases per 100,000 persons, making it a rare disease; however, its incidence and prevalence have been increasing recently [2, 3]. Regarding the treatment, intrasphincteric injection of botulinum toxin [4], balloon dilatation [5], surgical myotomy [6], and oral endoscopic myotomy (POEM) [7] are performed to release the functional obstruction at the LES level, but these procedures are all merely coping strategies. The cause of achalasia has been suggested to be autoimmune disease [8, 9], viral infections [10, 11], and allergic diseases [12, 13], but the cause has not yet been elucidated. The reason why the cause of achalasia has yet to be elucidated, despite various studies to date, may be due to its mode of onset and long-term course of the disease leading up to diagnosis. Achalasia generally has a insidious onset with gradual damage to LES neurons [14, 15]. Given that the sphincter loses its ability to relax over time [15], the underlying cause of LES neuron damage may have disappeared by the time of diagnosis. Therefore, the present study aimed to examine in detail the initial symptoms and the course of symptoms and to find new insights into the cause and course of the disease.

## Materials and methods

### Design and patients

This single-center, retrospective cohort study was conducted according to the Strengthening the Reporting of Observational Studies in Epidemiology (STROBE) guidelines [16]. Among the 804 patients who underwent high-resolution manometry (HRM) testing at Chiba University Hospital between March 2011 and August 2022, we enrolled 136 patients diagnosed with achalasia. This study was approved by the Chiba University Ethics Committee (approval number 4063).

### Questionnaire

The questionnaires were mailed to all patients. The questionnaire included information on medical history, allergies, medications, age at onset, initial symptoms, type of onset (acute or insidious onset), and age at the time of diagnosis, as well as a space for free answers. All patients completed the frequency scale for the symptoms of GERD (FSSG) questionnaire before HRM testing. The FSSG questionnaire consists of 12 items categorized into two domains, with each item scored from 0 (never) to 4 (always). Dysmotility-like symptoms (dyspepsia score), the first domain, are calculated by summing the scores (range: 0–28) for items 1, 4, 6, 7, 9, 10, and 12. Acid reflux-related symptoms (reflux score), the second domain, involved summing the scores (range: 0–20) for items 2, 3, 5, 8, and 11 [17]. Acute onset was defined as a sudden onset of symptoms with a definite date and time of onset; otherwise, it was defined as an insidious onset.

#### HRM

The HRM system used was the Diversatek system (Boulder, Colorado, USA) with 32 pressure and 16 impedance sensors. Patients were asked to swallow 5 mL of water 10 times in the supine or semi-Fowler's position. Achalasia was diagnosed according to the Chicago Classification; version 4.0 was used for diagnosis after 2021 and version 3.0 before that date [18, 19]. Resting LES pressure was defined as LES pressure for 30 s without swallowing.

### Endoscopic findings

All patients underwent upper gastrointestinal endoscopy. For diagnosing eosinophilic esophagitis (EoE), esophageal multiple mucosal biopsies were performed with or without endoscopic findings, and EoE was diagnosed if the eosinophilic infiltrate was ≥ 15/HPF [20].

### Statistical analysis

Baseline data are presented as the median and interquartile range. The data were analyzed using the GraphPad Prism software (version 8; Massachusetts, USA). Comparisons of the two groups were performed using Mann–Whitney U tests. The correlation coefficient was calculated using Spearman’s rank correlation coefficient. Differences with *p* < 0.05 were considered significant. For comparisons among the three groups, the significance level was set at 0.017 (0.05/3) and multiplicity was adjusted using the Bonferroni method.

## Results

### Study flow diagram and patient demographics

**Fig. 1:**
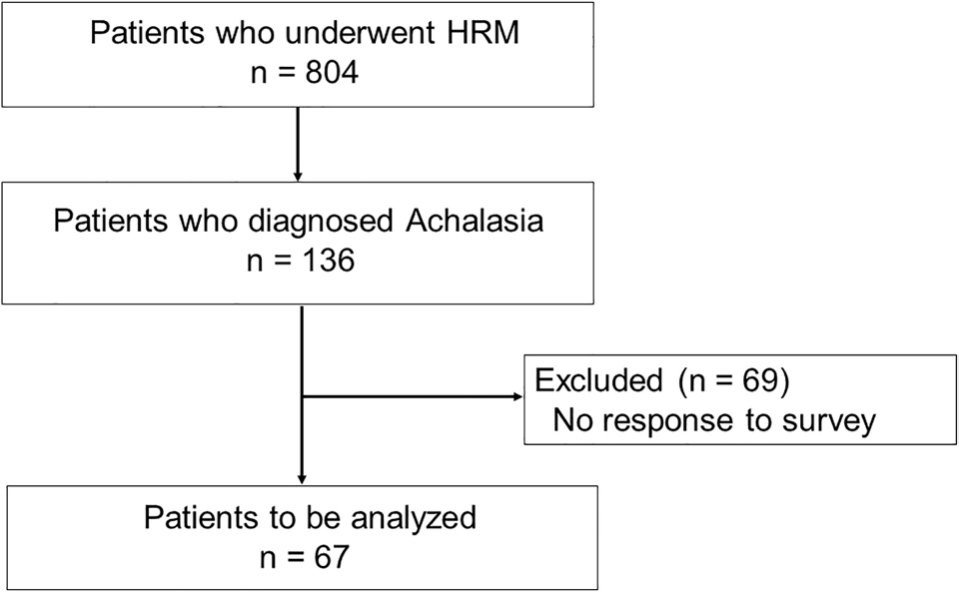
Flowchart of patient enrolment. HRM: high-resolution manometry

Figure 1 shows the flowchart of patient enrolment. In total, 67 (49.3%) of 136 patients with esophageal achalasia responded to the questionnaire. The patients’ background characteristics are shown in Table 1. There were 31 males and 36 females, and the median age of diagnosis of 58 years. Regarding the medical history, two cases had EoE (out of 31 patients who underwent biopsy) and one had scleroderma. No patient had a family history. The median age of onset was 42 years.

**Table 1:** Clinical characteristics of patients with achalasia

Clinical characteristics	n = 67
Sex (male/female)	31/36
Age at onset (years), median (IQR)	42 (31.8–57.8)
Age at diagnosis (years), median (IQR)	58 (41.3–73.3)
Comorbidity	
Hypertension, n (%)	15 (22.7%)
Diabetes mellitus, n (%)	5 (7.5%)
Eosinophilic esophagitis, n* (%)	2 (6.1%)
Scleroderma, n (%)	1 (1.5%)
Anhidrosis, n (%)	3 (4.5%)
Allergy, n (%)	47 (70.1%)
Asthma	5 (7.5%)
Atopic dermatitis	7 (10.4%)
Pollen allergy	23 (34.3%)
Food/Drug allergy	10 (14.9%)
Other (e.g., animals)	2 (3.0%)
HRM results	
Type I Achalasia, n (%)	38 (56.7%)
Type II Achalasia, n (%)	23 (34.8%)
Type III Achalasia, n (%)	6 (9.1%)
LES resting pressure, median (IQR, mmHg)	43 (31–54.3)
IRP, median (IQR, mmHg)	38 (30.6–44.1)

Data are presented as n (%)

*HRM* high-resolution manometry, *IRP* integrated relaxation pressure, *LES* lower esophageal sphincter, *IQR* interquartile range

*Of the 31 cases in which esophageal biopsies were performed

### Time to diagnosis

**Fig. 2:**
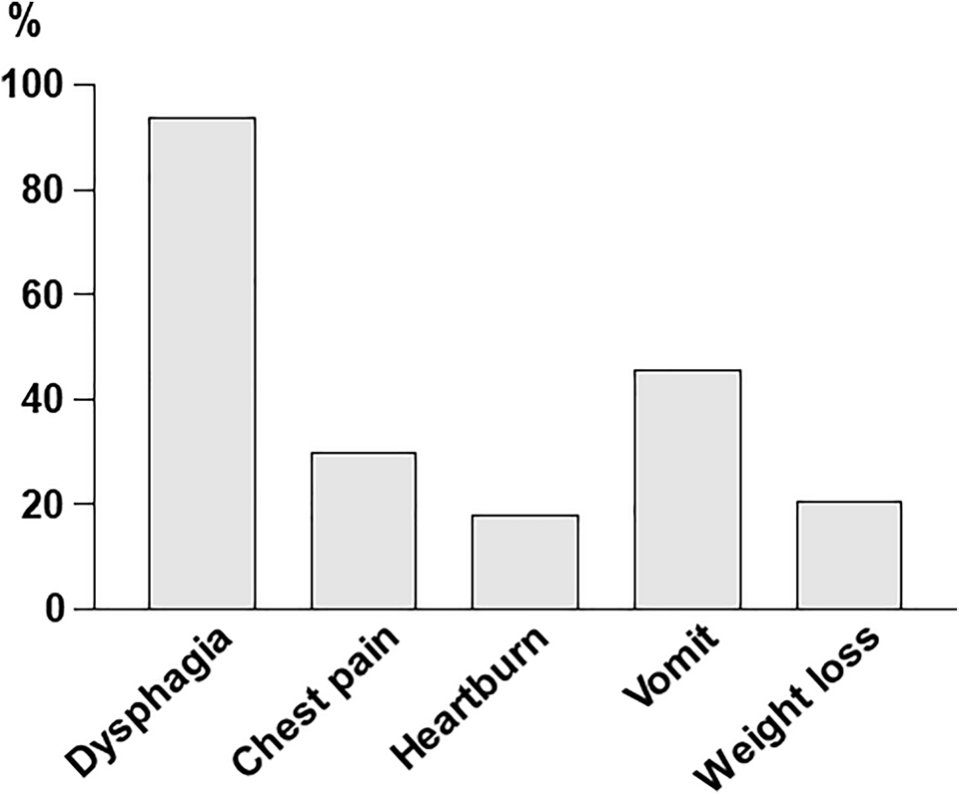
Symptoms at the first disease onset

**Fig. 3:**
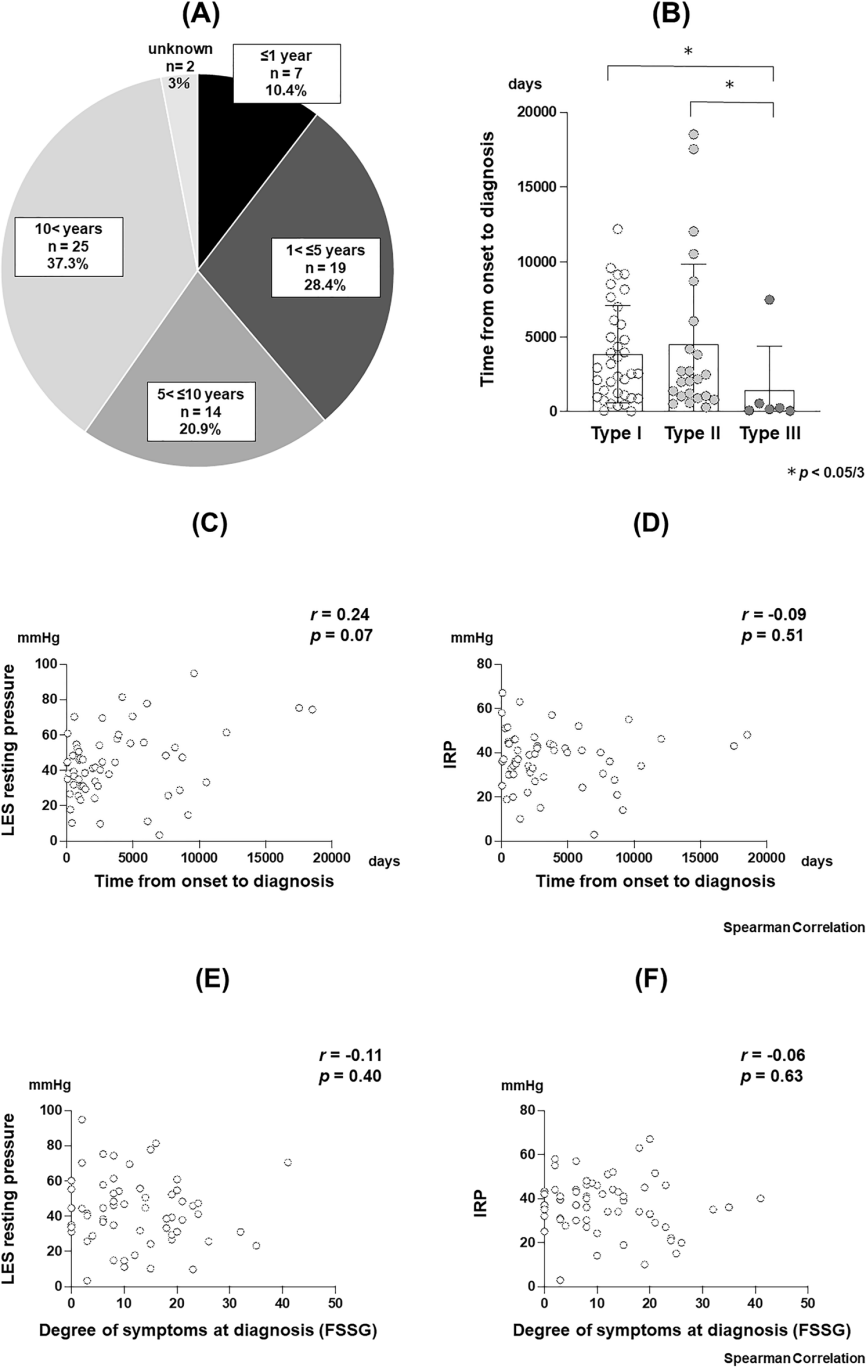
**A** Time from onset to diagnosis. **B** Time from onset to diagnosis by achalasia type. **C** Correlation between the time from onset to diagnosis and LES pressure at the time of diagnosis. **D** Correlation between the time from onset to diagnosis and IRP at the time of diagnosis. **E** Correlation between degree of symptoms and LES pressure at the time of diagnosis. **F** Correlation between degree of symptoms and IRP at the time of diagnosis. LES, lower esophageal sphincter; IRP, integrated relaxation pressure; FSSG, the frequency scale for the symptoms of GERD

Dysphagia accounted for 90% of the initial symptoms, with other symptoms varying from chest pain, heartburn, vomiting, and weight loss (Fig. 2). The median age at the time of diagnosis was 58 years, and the time from onset to diagnosis was 78.6 months. Altogether, 10.4% and 37.3% of the cases were diagnosed within 1 year of onset and > 10 years later, respectively (Fig. 3A). There were two patients who could not provide a definitive answer regarding the time of onset (Fig. 3A).

### Differences by achalasia type

The median time from onset to diagnosis by achalasia type was 91.3 months for Type I, 72.5 months for Type II, and 7.1 months for Type III. Type III cases were diagnosed significantly earlier than Type I and II cases (*p* = 0.014, *p* = 0.006) (Fig. 3B). Correlation analyses of HRM results (LES resting pressure, IRP) at diagnosis and time from onset to diagnosis are shown in Figs. 3C and D. Neither LES resting pressure (*r* = 0.24, *p* = 0.07) nor IRP value (*r* = − 0.09, *p* = 0.51) correlated with time to diagnosis. (Figs. 3C and D). Correlation analyses between HRM results (LES resting pressure, IRP) at diagnosis and degree of symptoms are shown in Figs. 3E and F. No correlation was found between HRM results and symptom severity (Figs. 3E and F).

### Onset types of achalasia

**Fig. 4:**
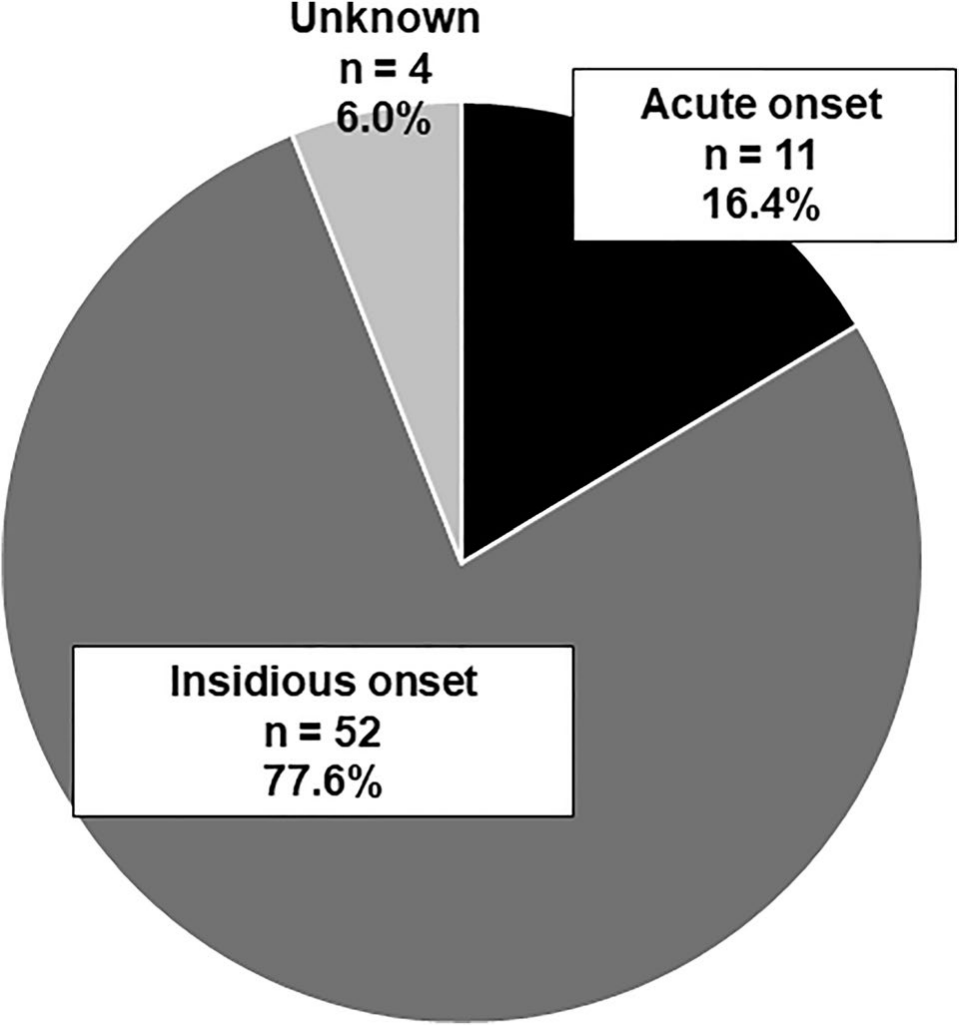
Pattern of the beginning of symptoms

Results for type of onset are shown in Fig. 4. There were two types of onsets: acute onset in 16.4% of patients and insidious onset in 77.6%. Four patients were unable to provide a clear answer regarding the type of onset (Fig. 4). Three patients (27.3%) had anhidrosis in the acute onset group; two of the three patients were subsequently examined by a neurologist and diagnosed with postacute onset autonomic neuropathy, with one of them receiving steroid pulse therapy, but the effect was inadequate.

## Discussion

The present study examined the form of onset of achalasia and the process leading to the diagnosis of the disease. Achalasia is considered a slowly progressive disease [1]. However, after a detailed survey using questionnaires, we found that there are acute-onset cases of achalasia. Additionally, several cases with acute-onset type also had anhidrosis at the same time. In the present study, we conducted a detailed investigation from the onset of symptoms to diagnosis. The results revealed that there were differences in the types of onsets of symptoms and that it took a long time to reach a diagnosis, with a median time of 78 months. Additionally, some patients were suspected of having a psychiatric disorder and were seen by multiple departments, including a psychiatrist. The existence of an acute-onset type of achalasia has been reported in recent years [21, 22]. Both reports were of onset after COVID-19 infection, and acute-onset achalasia may have been due to viral infection.

Anhidrosis is a disorder in which an individual does not sweat even in an environment that promotes sweating (high temperature and humidity) [23]. There are congenital and acquired forms of anhidrosis, and the causes of acquired generalized anhidrosis are classified into secondary sweating disorders caused by abnormalities of the eccrine sweat glands and sympathetic nerves, drugs, acquired idiopathic generalized anhidrosis, and autoimmune diseases such as autoimmune autonomic ganglionopathy [24]. Two cases of anhidrosis in this study were diagnosed as autoimmune autonomic ganglionopathy. Autoimmune autonomic ganglionopathy is also an autoimmune disorder of unknown etiology, characterized by a variety of autonomic nervous system symptoms such as orthostatic hypotension, urinary retention, and sweating abnormalities. Digestive symptoms such as constipation and achalasia have been reported as complications. It has been reported that 14% of patients with autoimmune autonomic ganglionopathy had prodromal symptoms, such as flu-like symptoms, shortly before the onset of autonomic symptoms [25]. This may support an association between acute-onset achalasia and viral infection, including associated autonomic symptoms. One of these patients was referred to a neurologist who diagnosed the patient with a sequela of acute autonomic neuropathy. Subsequently (approximately 1 month after disease onset), steroid pulse therapy was performed, but no improvement was seen, probably because a long time had elapsed between onset and treatment. However, the present results suggest that there may be cases of esophageal achalasia, especially with autonomic neuropathy, for which medical treatment is effective. Early therapeutic intervention may have the potential to improve symptoms. Furthermore, it is also known that there are cases of pseudo-achalasia secondary to malignant disease when patients have acute onset of symptoms [26]. Considering this point, patients with acute onset of symptoms should be examined quickly.

Most coherent reports of achalasia have come from Europe and the United States [27, 28]. In Japan, Sato et al. reported 385 cases of achalasia nationwide, with a prevalence rate of 7.0 per 100,000 persons, similar to that reported in Western countries [29]. The types of disease onset and time to diagnosis examined in the present study have not been investigated. Regarding the time from symptom onset to diagnosis, Niebisch et al. reported an average of 25 months (approximately 2 years) from symptom onset to diagnosis in Germany [30]. They noted that early achalasia is difficult to diagnose by endoscopy alone and that the widespread use of HRM is useful for early diagnosis. Eckardt et al. also studied 87 German cases of achalasia and concluded that the mean symptom duration was 4.7 ± 6.4 years, and that the delay in diagnosis was not due to atypical clinical symptoms but rather to the misinterpretation of typical findings by the consulting physician [31]. Contrarily, our cohort took a median of 78 months (approximately 6.5 years), which is longer than that reported in the German study [30, 31]. This difference may be due to the low application rate of HRM in Japan, which may have affected the time to diagnosis.

The present study has several strengths. First, it is the first report summarizing the two symptoms onset types of achalasia. Second, a detailed survey of patients, rather than a chart review alone, was conducted. Given that it is difficult to conduct a detailed survey based on chart review alone, a questionnaire was mailed along with it and a survey was also conducted in this study. Third, this is the first Asian report describing the time from disease onset to the confirmation of diagnosis. Despite its strengths, this study has several limitations. First, the questionnaire collection rate was low, and not all cases could be investigated. The unresponsive patients were excluded from the study because the information obtained from the chart review alone was not accurate. Second, the sample size, especially for Type III achalasia and anhidrosis, was small. Therefore, results may differ if a large number of cases is included. Third, biopsies for the diagnosis of EoE were not performed in all cases, but in about half. Therefore, this result may contain bias. Fourth, it has been reported in recent years that achalasia without high IPR values exists [32]. The IRP values of the cases included in this study were relatively high (median IRP 38 [30.6–44.1] mm Hg), which may have influenced the correlation between IRP values and time to diagnosis. Fifth, the present work was a single-center, retrospective study; hence, a further study with a larger number of patients at multiple centers are warranted to determine whether similar results can be obtained.

In conclusion, the early diagnosis of achalasia is important for the early improvement of patients’ quality of life and for health economics reasons. Our new findings may help to elucidate the pathogenesis of achalasia in the future.

## Data Availability

The data that support the findings of this study are available from the corresponding author, TM (ORCID, 0000-0001-5314-9325), upon reasonable request.
